# A ubiquitin-like domain is required for stabilizing the N-terminal ATPase module of human SMCHD1

**DOI:** 10.1038/s42003-019-0499-y

**Published:** 2019-07-10

**Authors:** Lars C. Pedersen, Kaoru Inoue, Susan Kim, Lalith Perera, Natalie D. Shaw

**Affiliations:** 1 Genome Integrity and Structural Biology Laboratory, National Institute of Environmental Health Sciences, National Institutes of Health, Research Triangle Park, NC 27709 USA; 2 Pediatric Neuroendocrinology Group, Clinical Research Branch, National Institute of Environmental Health Sciences, National Institutes of Health, Research Triangle Park, NC 27709 USA; 3 Reproductive Endocrine Unit, Massachusetts General Hospital, Boston, MA 02114 USA

**Keywords:** X-ray crystallography, Hydrolases, Neuromuscular disease

## Abstract

Variants in the gene *SMCHD1*, which encodes an epigenetic repressor, have been linked to both congenital arhinia and a late-onset form of muscular dystrophy called facioscapulohumeral muscular dystrophy type 2 (FSHD2). This suggests that SMCHD1 has a diversity of functions in both developmental time and space. The C-terminal end of SMCHD1 contains an SMC-hinge domain which mediates homodimerization and chromatin association, whereas the molecular architecture of the N-terminal region, which harbors the GHKL-ATPase domain, is not well understood. We present the crystal structure of the human SMCHD1 N-terminal ATPase module bound to ATP as a functional dimer. The dimer is stabilized by a novel N-terminal ubiquitin-like fold and by a downstream transducer domain. While disease variants map to what appear to be critical interdomain/intermolecular interfaces, only the FSHD2-specific mutant constructs we tested consistently abolish ATPase activity and/or dimerization. These data suggest that the full functional profile of SMCHD1 has yet to be determined.

## Abstract (web summary)

Lars Pedersen, Kaoru Inoue et al. present the crystal structure of the N-terminal ATPase module of human SMCHD1 bound to ATP. They show that the dimer is stabilized by a ubiquitin-like fold at the N-terminal, as well as by a downstream transducer domain.

## Introduction

The GHKL (gyrase, Hsp90, histidine kinase, and MutL) superfamily of ATPases/kinases includes a group of functionally unrelated proteins unified by the presence of an unconventional ATP-binding fold (the Bergerat fold) at their N-terminal^1–3^. This fold includes a GHKL-ATPase core domain and an adjacent α/ß domain referred to as the transducer domain and formerly, the S5 domain^1^. The GHKL core contains a central, cone-shaped ATP-binding pocket that is delimited by 4–6 α-helices, the transducer (from below), and by a long, flexible loop termed the ATP-lid^1^. Four sequence motifs which line the ATP binding site, and a fifth motif in the transducer, contain key catalytic residues and are highly conserved among the GHL subclass of GHKL-ATPases. In most GHL proteins, the GHL/transducer domain is linked to a C-terminal domain that mediates constitutive homodimerization^3^.

Structural studies in GHKL family members have demonstrated that dynamic conformational changes occur at the N-terminal end upon ATP binding and hydrolysis. These include stabilization and closure of the ATP lid, rotation of the transducer domain into the GHKL core, and finally, N-terminal dimerization that is coordinated by a pre-N-domain (‘strap’) swap between protomers. Simultaneous dimerization at the N-terminal and C-terminal ends thereby forms a molecular clamp around a DNA or peptide client whereby release of the clamp is coupled to ATP hydrolysis^1^.

**Fig. 1:**
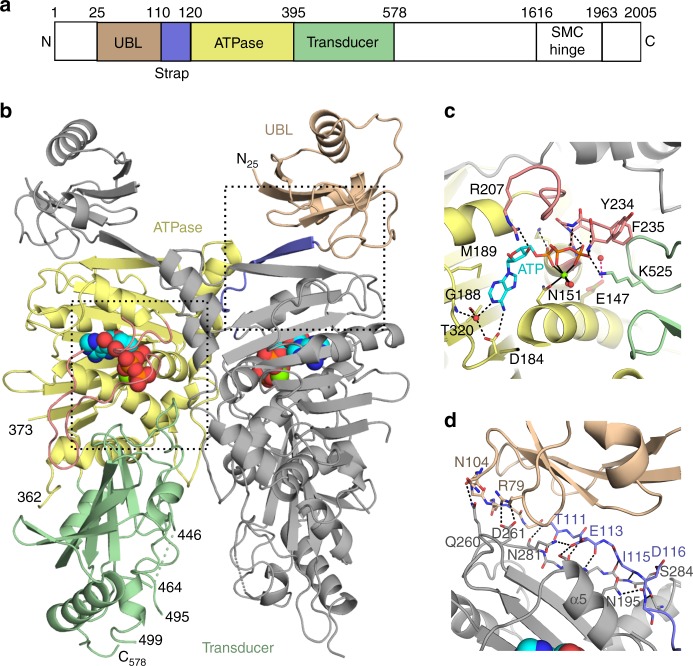
Dimeric structure of the N-terminal region of the SMCHD1 ATPase module. **a** Domain organization of full-length SMCHD1. **b** Cartoon of the crystal structure of the ATPase module (24–578aa). Residues in the pre-N ubiquitin-like domain (25–109aa), strap (110–120aa), GHKL-ATPase domain (121–395aa), and transducer domains (396–578aa) are colored wheat, lavender, pale-yellow and pale-green, respectively. The ATP molecule is colored cyan. **c** ATP binding to the active site. General location in the dimer is outlined with the bottom left box in panel **b**. ATP is shown in stick form alongside the catalytic Mg^2+^ (green) and coordinating water molecules (red spheres). Secondary structural elements are colored as in panels **a** and **b**. Hydrogen bonds to the ATP molecule and coordination interactions with the Mg^2+^ ion are shown as dashed and solid black lines, respectively. The catalytic glutamate (Glu147, transparent olive) has been modeled into the active site using the crystal structure of MORC2. The proposed hydrogen bond between Glu147 and the nucleophilic water is shown as a dotted red line. Both the ATP-lid (rose) and Lys525 from the switch-loop of the transducer domain form hydrogen bonds with the ATP phosphates. **d** Dimerization interactions of the UBL domain and strap. General location in the dimer is outlined with upper right box in panel **b**. There are extensive hydrogen bonds (black dotted lines) between the strap of one monomer (lavender) and the GHKL-ATPase domain (gray) of the other protomer. Additional interactions between the UBL domain (wheat) of one monomer and the GHKL-ATPase domain of the other monomer may also contribute to dimerization as constructs starting at residue 110 (excluding the UBL domain) do not dimerize

Structural maintenance of chromosomes hinge-domain containing protein 1 (SMCHD1), an emerging player in the field of epigenetics, is a unique member of the GHKL superfamily. Originally identified in a mutagenesis screen for novel epigenetic repressors of repeat sequences^4^, SMCHD1 is now known to not only silence repeats but also a subset of autosomal genes, particularly those that are imprinted, mono-allelically expressed, or clustered^5^, and to maintain X-chromosome inactivation^5–7^. Like other GHKL family members, SMCHD1 contains a weak N-terminal GHKL-ATPase (111–365aa)^8,9^ and is predicted to contain an S5-fold/transducer domain^9,10^. At the C-terminal end lies an SMC-hinge domain (1616–1963aa) implicated in protein homodimerization (Fig. 1a)^9^. It is the non-conserved and uncharacterized regions, however, that account for the large size (≈230 kDa, 2005aa) of SMCHD1. The region immediately upstream of the GHKL domain, for example, has not yet been investigated, but in related proteins (e.g. GRP94, the Hsp90 cytosolic paralog), this pre-N terminal domain regulates ATP hydrolysis and dimer closure^11,12^. SMCHD1 has also been predicted to contain a bromo-adjacent homology-like domain and a short coiled-coil domain^9,10^. Deletion of the predicted bromo-adjacent homology-like domain prevents SMCHD1 localization to the inactive X-chromosome with no additional functional consequences^9^. Although it has been hypothesized that SMCHD1 may form a molecular clamp akin to other GHKL family members^13,14^, previous studies reported that an SMCHD1 N-terminal construct (111–702aa), while capable of ATP hydrolysis, was entirely monomeric^8^.

*SMCHD1* has recently garnered attention because variants in this gene cause two distinct disorders: fascioscapulohumeral muscular dystrophy type 2 (FSHD2), a rare, late-onset form of muscular dystrophy^15^, and congenital arhinia^16,17^, a severe craniofacial malformation. Although the spectrum of *SMCHD1* variants in patients with arhinia and FSHD2 are not completely distinct, in FSHD2, variants are more evenly distributed across the entire 48-exon gene, whereas in arhinia, variants are tightly clustered in the N-terminal region (107–552aa) encompassing the extended ATPase domain^16,17^. This restricted localization suggests that aberrant ATPase activity may be a critical part of disease pathogenesis in arhinia, yet none of the disease variants map to the critical, conserved residues of the ATP-binding fold. Further, previous studies of ATP hydrolysis in recombinant Smchd1 (111–702aa) did not demonstrate consistency of direction among 12 arhinia-specific variants^16,18^. Given the dynamic conformational changes that have been described in the N-terminal region of other GHKL ATPases, it is quite possible that these variants disrupt ATP-dependent conformational changes in SMCHD1 rather than ATP hydrolysis.

Here we report the crystal structure of the human SMCHD1 N-terminal GHKL-ATPase/transducer module. We find that like other GHKL-ATPases, SMCHD1 contains three conserved catalytic residues that are required for ATP hydrolysis: Asn151 (Motif I) coordinates the metal ion, Glu147 (Motif I) activates the water molecule for hydrolysis, and K525 (Motif V, transducer domain) regulates catalysis. The N-terminal construct also demonstrates ATP-dependent dimerization. The dimer is stabilized by a pre-N-domain swap that includes the strap and a unique ubiquitin-like (UBL) fold. Dimerization generates a deep cavity between two disordered negatively charged loops of the transducer domains that may reasonably accommodate a cationic client. Arhinia and FSHD2 variants map to the protein surface, dimer and domain interfaces, hydrophobic pockets, and near the ATP binding site. Several FSHD2 variants abolish ATPase activity and preclude dimerization, whereas the arhinia variants studied have only modest effects on catalysis and do not disrupt dimerization. Disease variants may therefore disrupt a novel protein function, protein interaction, conformational change, and/or a delicate monomer/dimer equilibrium that regulates the repressor function of SMCHD1.

## Results

### Overall structure

**Fig. 2:**
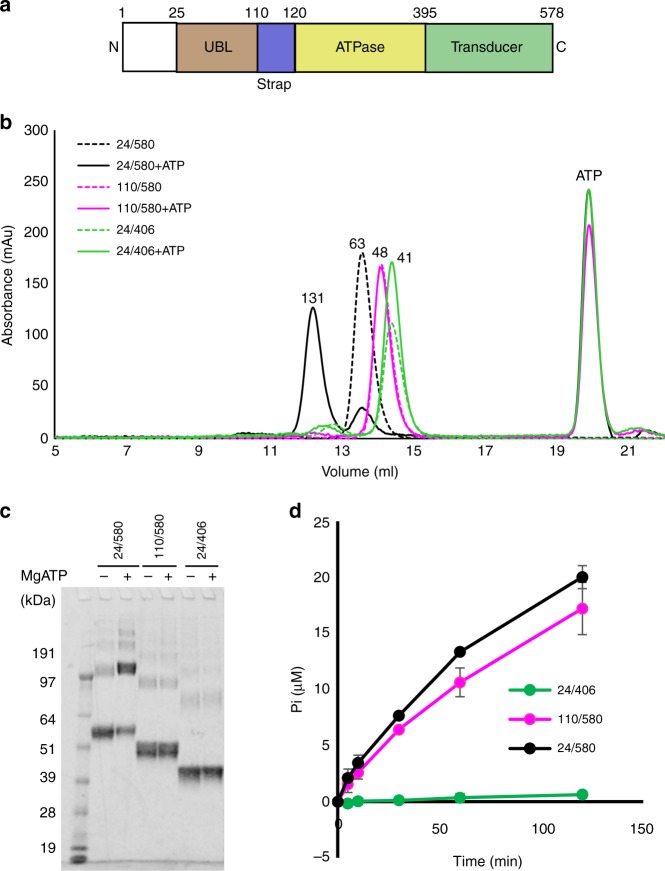
Biochemical characterization of SMCHD1 N-terminal constructs. **a** Domain organization of SMCHD1 N-terminal ATPase module colored as in Fig. 1. **b** A shift from monomer to dimer in the presence of 5 mM Mg^2+^/ATP is shown for the 24–580 construct. Molecular weights above the peaks are estimates based on elution volumes of standards and are given in kDa. The theoretical molecular weights for the constructs are as follows: 24–406 (44.2 kDa), 110–580 (54.7 kDa), and 24–580 (64.5 kDa). **c** Dimerization by chemical crosslinking of SMCHD1 constructs (with glutaraldehyde) in the absence (−) and presence (+) of 5 mM Mg^2+^/ATP demonstrates substantial dimerization only in the 24–580 construct (129 kDa). **d** Radiometric ATPase assay in the three SMCHD1 constructs demonstrates comparable activity for 110–580 and 24–580 but minimal activity for 24–406. Measurements were taken at times 0, 5, 10, 30, 60, and 120 min with at least two biological replicates and are reported as mean ± sd. (Where not obvious, the sd is included in the symbol.)

The crystal structure of the GHKL-ATPase module in the presence of ATP was solved at 2.2 Å by expressing a construct consisting of residues 24–580 with a E147A inactivating variant (Fig. 1a, b, Supplementary Figs. 1 and 2, Table 1). The asymmetric unit contains two dimers which adopt similar conformations (RMSD of 0.71 Å over 972 Cαs). The monomers also show a high degree of structural similarity (Supplementary Fig. 3). Each monomer is comprised of three domains: a pre-N terminal domain with a ubiquitin-like fold (UBL) (25–109aa), a GHKL-ATPase catalytic domain (110–395aa), and a transducer domain (396–578aa). The previously uncharacterized UBL domain appears to be important for dimerization as its removal results in a catalytically competent enzyme incapable of forming dimers (Fig. 2). Although SMCHD1 has traditionally been classified as an atypical member of the SMC family of proteins, the overall structure reveals greater similarity to the MORC family of gene repressors^19,20^.

**Table 1:** Data collection and refinement statistics

	Refinement	Phasing
*Data collection*		
Space group	P2_1_2_1_2_1_	P2_1_2_1_2_1_
Cell dimensions		
* a*, *b*, *c* (Å)	108.76, 148.01, 191.14	108.76, 148.01, 191.14
*α*, *β*, *γ* (°)	90, 90, 90	90, 90, 90
Resolution (Å)	50.00–2.2	50.00–2.2
*R*_sym_	0.12 (0.80)^a^	0.13 (0.93)
*I* / σ*I*	16.8 (1.2)	22.5 (1.8)
Completeness (%)	97.4 (84.2)	96.6 (72.1)
Reflections	1,403,332	2,744,959
Redundancy	9.1 (7.0)	17.7 (11.3)
Post AC completeness^b^		86.7 (57.4)
# of Se sites found		19
*Refinement*		
Resolution (Å)	50.00–2.20	
# Unique reflections	152,943	
*R*_work/_ *R*_free_	0.196/0.230	
No. atoms	17,300	
Protein	16,235	
Ligand (ATP)	124	
Solvent	941	
*B*-factors		
Protein	48.3	
Ligand (ATP)	28.5	
Solvent	44.73	
R.m.s. deviations		
Bond lengths (Å)	0.004	
Bond angles (°)	0.664	
Ramachandran (%)		
Favored	97.05	
Allowed	2.85	
Outliers	0.10	

λ used for this study was 0.97625 Å. The two data sets represent different processing of the data collected from multiple scans of the same single crystal

^a^Values in parentheses are for highest-resolution shell

^b^Completeness after truncation of weak data using Auto Correction option in HKL2000

### Catalytic domain

The SMCHD1 GHKL-ATPase catalytic domain (110–395aa) consists of a Bergerat ATP-binding fold defined by Motifs I–IV (Supplementary Fig. 1)^2,3,21^. ATP bound to the active site is stabilized by interactions with residues from each of these motifs and by an octahedrally coordinated Mg^2+^ ion that forms interactions with oxygens from each of the three phosphate groups of ATP and with the conserved Asn151 from Motif I on α-helix 4 (Fig. 1c). The nucleotide base is positioned in a pocket surrounded by residues from Motifs I, II, and IV. The proposed catalytic base, Glu147 (mutated to Ala in the crystal structure to inhibit ATP hydrolysis), is also located within Motif I on the same central α-helix as Asn151. Modeling the Glu sidechain based on the position of the equivalent Glu in the MORC2 structure bound to AMPPNP^20^ positions the Glu sidechain within hydrogen bonding distance of the likely nucleophilic water molecule (Fig. 1c). Aspartate 184, found in the conserved DNGXG of Motif II at the end of the third β-strand in the central ATPase fold (β7 in SMCHD1, Supplementary Fig. 1), forms a hydrogen bond with the N6 atom of the adenine base (Fig. 1c). This interaction, along with water-mediated interactions between the N1 atom of ATP and residues Asp184 and Gly188, enforce specificity for ATP over other nucleotides^1^. In GHKL-ATPases, ATP binding triggers a conformational change in the ATP lid^21–24^. In the SMCHD1 crystal structure, the ATP lid exists in a closed conformation and is located between Motif II and Motif III and extends into Motif III. Arginine 207 within the lid interacts with the 3′OH of the ATP molecule, and backbone amides from residues Tyr234-Gly238 of Motif III interact with the γ-phosphate, effectively cradling ATP in place (Fig. 1c). Motif IV is adjacent to Motif II at the N-terminus of β-strand 11 (Fig. 1c and Supplementary Fig. 1) and contains a conserved Thr, Thr320, that also helps to define the adenine base binding pocket.

### Transducer domain

The transducer domain (396–578aa) contains a six-stranded mixed β-sheet and forms an interface with the ATPase domain and with the ATP-lid that are both stabilized by hydrophobic and hydrophilic interactions. A loop between β-strand 17 and α-helix 15, termed the “switch-loop”, extends from the central domain structure along the dimer interface and positions Lys525 at the ATP binding site for a hydrogen bond with the γ-phosphate (Fig. 1b, c, Supplementary Fig. 1). This Lys, termed the “switch” lysine, represents a fifth conserved Motif found in the GHL subclass of GHKL-ATPases. It is proposed to sense nucleotide binding, induce conformational changes associated with monomer/dimer interconversion^1,25,26^, and stabilize the pentavalent transition state of the γ-phosphate during hydrolysis^21,27,28^. The SMCHD1(24–406) construct, which lacks the transducer domain, does not dimerize and has diminished ATPase activity, suggesting the transducer domain plays a role in both dimerization and catalysis (Fig. 2).

### Dimerization and potential clients

The SMCHD1(24–580/E147A) construct forms the canonical GHKL-ATPase dimer with an N-terminal β-strand (110–120aa) “strap” that is swapped between monomers^27,29^. The strap extends the central β-sheet of the GHKL-ATPase domain (on the adjacent protomer) by capping the exposed edge of the sheet with an additional β-strand in an antiparallel configuration (Fig. 1a, b, d and Supplementary Fig. 1). In monomeric structures of TRAP1, the mitochondrial Hsp90, a similar strand is found in cis that is swapped upon dimerization^30,31^. It remains to be determined whether or not a similar cis arrangement exists in the monomer of SMCHD1. The dimer interface is also stabilized by interactions at residues 121–134, 191–206, 234–235, and 283–291 of the ATPase domain and residues 520–529 from the switch-loop of the transducer domain.

One unique feature of the N-terminal domain swap in SMCHD1 compared with other GHKL-ATPases is the addition of a UBL domain. The function of this domain is unknown. Addition of the UBL domain does not appear to affect the rate of ATP hydrolysis (Fig. 2d), but gel filtration and cross-linking experiments suggest that it contributes to dimerization as construct 24–580 dimerizes in the presence of ATP while construct 110–580 does not (Fig. 2b–c). Indeed, residues Arg79 and Asn104 in the UBL domain are well-positioned to form hydrogen bonds with Gln260 and Asp261 in the ATPase domain of the adjacent monomer (Fig. 1d).

**Fig. 3:**
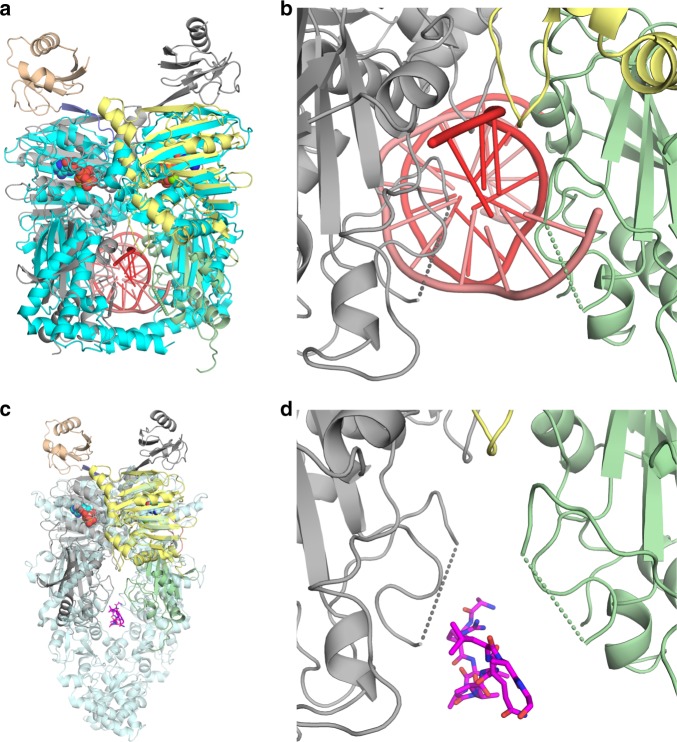
Superposition of SMCHD1 to GHKL-ATPases ParE44 and GRP94. **a** Superposition of topoisomerase ParE44 (cyan) with bound DNA (red) (pdbidcode:5J5Q; RMSD 3.5 Å, 484 Cα’s) to SMCHD1. **b** Close-up view of superimposed DNA (red) in the cavity of SMCHD1. The disordered Asp/Glu-loops from the transducer domains of SMCHD1 are represented by dotted lines. In the current conformation, these loops would likely form steric as well as electrostatic repulsive interactions with a negatively charged client like DNA. **c** Superposition of heat-shock protein GRP94 (transparent blue) with polypeptide (magenta) bound at the client-binding site (pdbidcode:5ULS, RMSD 4.0 Å, 541 Cα’s) to SMCHD1. **d** Close-up view of the superimposed client mimic peptide from GRP94 in the cavity of SMCHD1. SMCHD1 is colored as in Fig. 1

SMCHD1 N-terminal dimerization generates a cavity between the transducer domains of the two monomers that is flanked on both sides by a disordered loop (447–463aa). This loop contains a highly anionic region consisting of seven acidic Asp/Glu residues (Supplementary Fig. 1). Crystal structures of the bacterial topoisomerase ParE44 and the hsp90 paralog GRP94 reveal that this cavity can bind dsDNA or polypeptides, respectively^11,32^. However, superposition of SMCHD1 with the ParE44/DNA complex suggests that in the current conformation of SMCHD1, steric conflicts would exist between DNA and residues 135–136 of the ATPase domain and/or with residues 487–490 and 532–538 of the transducer domain (Fig. 3a, b). Further, electrostatic and steric clashes would be predicted between DNA and residues 441–468 which encompasses the disordered, highly anionic Asp-Glu region (447–463aa; Fig. 3b). Interestingly, superposition of SMCHD1 with GRP94 with a polypeptide bound in the cavity (Fig. 3c, d) suggests that in SMCHD1, a polypeptide client could be accommodated more easily than DNA, and if positively charged, such a polypeptide could possibly form favorable electrostatic interactions with the Asp/Glu-containing loop.

### Human disease variants

**Fig. 4:**
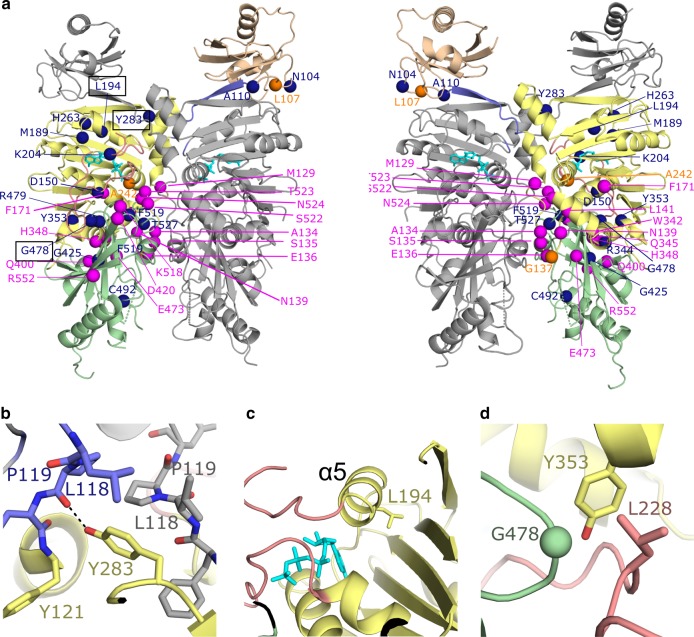
Location of variants associated with arhinia and FSHD2. **a** Cartoon diagram colored as in Fig. 1 with right panel ~180° rotation with respect to the left. Variants associated with arhinia, FSHD2, or with both diseases are colored magenta, dark blue, or orange, respectively. Some variants are only visible/labeled on one panel. **b**, **c**, **d** Close-up views of three regions in SMCHD1 where FSHD2 disease variants Y283C, L194F, and G478E (respectively) are located. The labels for these variants are boxed with black line on left figure of panel **a**. Black dashed lines indicate hydrogen bonds

All known arhinia and one-quarter of FSHD2 missense variants map to the ATPase module (Fig. 4a, Supplementary Fig. 1). These variants are clustered further in three regions of the primary sequence, and in the tertiary structure, most variants map to the ATPase/transducer domain or to dimer interfaces (Fig. 4, Supplementary Fig. 4). Based on conformational dynamics observed in other GHL proteins, it is possible that these variants interrupt critical conformational changes that lead to dimerization upon ATP binding and hydrolysis.

We studied the effect of 11 SMCHD1 variants implicated in either arhinia (p.S135C, p.N139H, p.Q345R, p.H348R, p.T523K, p.E473Q, and p.E473G), FSHD2 (p.L194F, p.Y283C, and p.G478E), or both (p.G137E) on protein stability, ATPase activity, and dimerization. Differential scanning fluorimetry (DSF) was used to analyze the thermostability of all constructs after purification by gel filtration. All mutant constructs displayed thermal melt curves consistent with a properly folded protein, and only two constructs, E473G and L194F, displayed shifts in Tm >2.5 °C (−4.9 and −2.8, respectively; Supplementary Fig. 5a, b, Supplementary Table 1). L194F also displayed increased initial fluorescence, possibly due to increased exposure of hydrophobic surfaces.

**Fig. 5:**
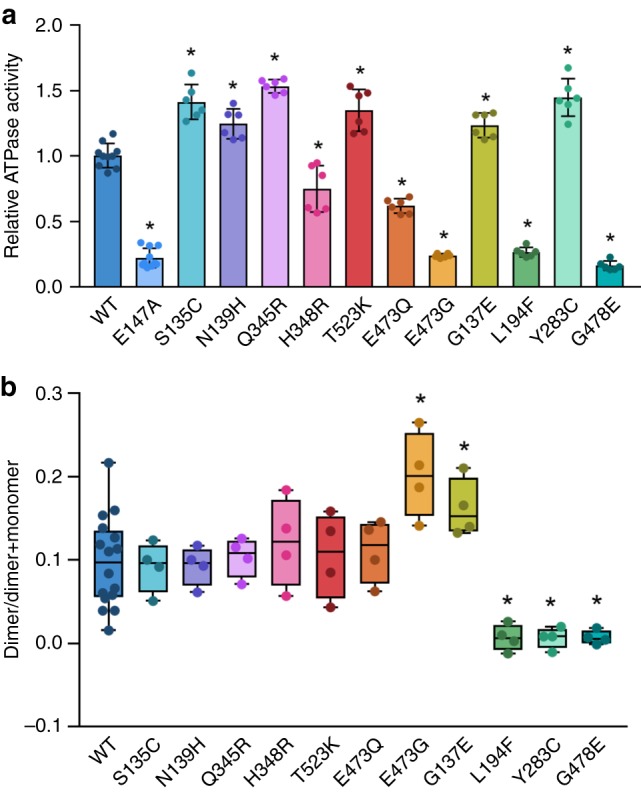
Catalytic activity (**a**) and dimerization capacity (**b**) in SMCHD1(24–580) wild-type (WT) and mutant constructs. **a** Arhinia and FSHD2 variants have variable effects on ATPase activity as determined by quantitation of released inorganic phosphate (γ-^32^_Pi = _γ-^32^P / γ-^32^P + [γ-^32^P]ATP). Data is presented as fold-change in activity (mean ± 1 SD) of mutant constructs relative to WT at the 60-min time point. The catalytically deficient mutant E147A is shown as a negative control. **b** Results of native PAGE assay demonstrating dimerization is intact in arhinia mutant constructs but diminished in FSHD2 mutant constructs. Data are presented in box-plot form where horizontal lines represent maximum, 75th percentile, median, 25th percentile, and minimum, respectively, from top to bottom. See Supplementary Fig. 6 for raw data. Data from both experiments reflects two technical replicates from two independent experiments. *P < 0.05 vs. WT

Most arhinia mutant constructs had K_M_, K_cat_, and V_max_ similar to that of wild-type (Supplementary Table 2). There were only modest increases or decreases in ATPase activity with the exception of one arhinia variant, E473G, which showed a >2-fold decrease in ATP hydrolysis (Fig. 5a). However, this change may reflect decreased protein stability of E473G as determined by DSF (Supplementary Table 1). Two of the three FSHD2 variants tested (L194F and G478E) also showed a >2-fold decrease in ATPase activity, whereas Y283C demonstrated slightly increased activity (Fig. 5a). The ATPase activity of eight of these variants has previously been tested in the form of a murine, monomeric Smchd1(111–702aa) construct^16,18^. Results of the current and previous ATPase assays are largely consistent in that all changes in activity are ≤3-fold. One exception is S135C which showed the greatest increase in activity (~2–5-fold) in the Smchd1(111–702aa) construct but only a modest increase (~1.3-fold) in our hands. The cause of this discrepancy is unclear but may be related to differences in the construct itself and/or ATPase assay conditions. Importantly, we did not observe a disease-specific signature of ATPase activity; bi-directional effects (increased or decreased ATPase activity) were observed in both arhinia and FSHD2 constructs.

In contrast, assays of dimerization (native PAGE and chemical cross-linking) revealed clear functional differences between arhinia and FSHD2 mutant constructs (Fig. 5b, Supplementary Figs. 6 and 7). Dimerization was preserved in all arhinia constructs but it was greatly diminished in all FSHD2 constructs tested (L194F, G478E, and Y283C). Of note, the absence of dimerization was not purely a result of diminished ATPase activity as Y283C displayed increased ATPase activity relative to wild-type. The crystal structure suggests several possible ways these mutations might interfere with dimerization. Tyrosine 283 from the ATPase domain is located at a critical position in the dimer interface (Fig. 4a, b). It forms a putative hydrogen bond with the carbonyl oxygen of “strap” residue Pro119 and is sandwiched between Tyr121 of its protomer and strap residues Leu118 and Pro119 of the other protomer in the dimer. Replacement of the Tyr residue with a Cys would disrupt hydrogen bonds and hydrophobic interactions. Leucine 194 is buried in a hydrophobic pocket on α-helix 5, on the opposite side of the ATP binding site (Fig. 4c). A variant at this position could destabilize the region between GHKL Motifs II and III which would affect catalytic activity and possibly expose more hydrophobic surfaces, providing an explanation for the increased initial fluorescence observed by DSF for the L194F mutant (Supplementary Fig. 5b). In addition, neighboring residues Ser191 and Asn195 from α-helix 5 form hydrogen bonds with the “strap” from the adjacent molecule (Fig. 1d). Therefore, alterations in the position of α-helix 5 could also interfere with the dimer associated “strap” interactions. Glycine 478 is located at the ATPase/transducer domain interface. A change at this position would likely disrupt the positioning of the ATP lid as Gly478 is packed tightly against residue Leu228 of the ATP lid immediately before Motif III (Fig. 4d). As the ATP lid is involved in ATP binding and interacts with domain and dimer interfaces, it is perhaps not surprising that variants such as L194F and G478E may disrupt lid positioning and consequently, eliminate ATP hydrolysis and dimerization.

## Discussion

SMCHD1 is a 2005aa protein with an N-terminal functional GHKL-ATPase module (1–578aa), an extended non-conserved middle region of unknown function, and a C-terminal SMC-hinge domain (1616–1963aa) that mediates constitutive dimerization. Herein, we have revealed the crystal structure of the SMCHD1 N-terminal module which contains all of the conserved elements of the Bergerat ATP-binding fold, a transducer domain, and a novel ubiquitin-like (UBL) fold. Contrary to previous reports, we demonstrate that the N-terminal module is indeed capable of ATP-dependent dimerization (Fig. 2b, c).

In family members of the GHL subset of GHKL-ATPases, ATP binding induces major conformational changes that optimally position the catalytic residues for ATP hydrolysis, stabilize the dimeric state, and generate a client-binding cavity. The switch-loop of the transducer domain, for example, which contains the Motif V Lys (or Arg), rotates upward and is inserted into the ATPase binding pocket, thereby positioning the switch Lys near the γ-phosphate^1^. The ATP lid between Motifs II and III closes down over the ATP molecule, and the N-terminal “strap” extends across to the neighboring molecule in the dimer. In hsp90s and topoisomerases, dimerization of the ATPase module also generates a central cavity that can bind client protein or DNA, respectively. As these proteins show only weak ATPase activity, ATP hydrolysis is believed to regulate the timing of state transitions as opposed to delivering a power stroke^1,13^. For example, results of stopped-flow experiments in Hsp90 are most consistent with a complex, five state on-pathway kinetic model for the ATPase cycle in which conformational changes are much slower than the ATP hydrolysis step itself^33^. At steady state conditions in the presence of ATP, the ATP-bound Hsp90 state is the most abundant, and the closed conformation is observed <5% of the time. When hydrolysis is slowed by replacing ATP with ATPγS, however, the closed Hsp90 state is observed 60% of the time. We similarly observed by native PAGE, that in the presence of ATP, the SMCHD1 N-terminal module exists as a dimer only ≈10% of the time. The structural and biochemical similarity between the SMCHD1 N-terminal module and the GHL family at large therefore suggests that SMCHD1 may also undergo a complex conformational cycle that temporally links ATP binding and/or hydrolysis to client binding. This hypothesis awaits further study.

**Fig. 6:**
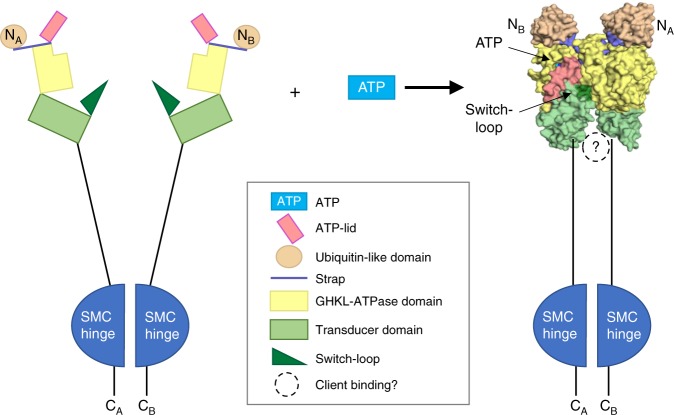
Proposed clamp-like closure of SMCHD1 upon ATP binding. Potential conformational changes in SMCHD1 during the transition from apo-monomer to ATP-bound dimer based on similar conformational changes that occur upon dimerization in other GHKL-ATPases. Binding of ATP results in closure of the ATP lid along the ATPase/transducer interface and repositioning of the transducer domain and switch-loop with respect to the ATPase domain; this also coincides with a domain swap of the strap and ubiquitin-like (UBL) domain. In other GHKL-ATPases, dimerization generates a binding cavity at the C-terminal end of the transducer domain. This cavity may serve as a binding site for SMCHD1 binding partners

Dimerization of the SMCHD1 N-terminal module may trap a client in the central cavity between the N-terminal ATPase/transducer module (the “jaws” of the clamp) and the downstream SMC-hinge domain (Fig. 6)^14^. Negative stain electron microscopy of full-length SMCHD1 indeed suggests that SMCHD1 forms barbell-like homodimers with globular domains at both the N- and C-termini^9^. In the crystal structure of the dimeric SMCHD1 N-terminal module, the central cavity is lined with a disordered loop containing seven Asp/Glu residues. Based on superpositions, this cavity is likely to be too small and/or too anionic to bind DNA but might accommodate a cationic polypeptide from a positively charged histone (Fig. 3). Asp/Glu-rich sequences of this sort have been proposed to modulate gene expression through DNA or RNA mimicry and/or by interacting with histones and transcription factors^34^. In DNA methyltransferase-I (DNMT1), for example, a long, acidic segment called the autoinhibitory linker connects the DNA-binding (CXXC) domain and the bromo-adjacent homology-like 1 domain. This linker repels unmethylated DNA from the enzyme active site, ensuring that only hemimethylated DNA is a substrate for maintenance methylation^35^.

SMCHD1 has a novel N-terminal ubiquitin-like (UBL) fold that does not appear to modulate ATPase activity but that is required for GHKL-ATPase module dimerization (Fig. 2). The two organellular hsp90s, GRP94 (endoplasmic reticulum) and TRAP1 (mitochondria), also contain a pre-N domain whose function has been well-characterized. In both GRP94 and TRAP1, the pre-N domain suppresses ATPase hydrolysis^11,31^, and in GRP94, the particularly long pre-N domain (residues 22–72) also appears to suppress dimer formation and to participate in client maturation. Given their sequestration in organelles, however, GPR94 and TRAP1 may have evolved a complex pre-N domain to replace the role of hsp90 co-regulators that are only present in the cytoplasm^11^. Thus, the pre-N UBL of SMCHD1, which is a nuclear protein, may serve a novel functional role and this question is now under investigation.

The current studies suggest that differences in SMCHD1 ATPase activity alone do not explain the divergent phenotypes of FSHD2 and arhinia. Several N-terminal variants found in these conditions fall outside the ATPase/transducer domain (at residues 104, 107, and 110), and those that do occur within the catalytic component spare all of the conserved residues in the Motifs that define the Bergerat ATP-binding fold as well as the Motif V Lys in the transducer domain^2,3^. Further, mutant constructs from FSHD2 and arhinia demonstrate a high degree of overlap in their effect on SMCHD1 ATPase activity both in terms of direction and amplitude. In contrast, disruption of dimerization was specific to the FSHD2 variant constructs tested and thus provides one possible molecular mechanism underlying phenotypic divergence in FSHD2 and arhinia.

Importantly, however, ultra-rare missense variants at identical amino acid residues (107, 137, and 242) have now been identified in patients with arhinia and patients with FSHD2^36^. The finding that the same variant can cause either condition suggests a more complex genetic paradigm where phenotypic differences arise from variation in prenatal exposures, SMCHD1 binding partners, or SMCHD1 downstream targets rather than from differences in a single biological function. Although the precise functional effects of these SMCHD1 missense variants remain to be determined, the discovery of SMCHD1 N-terminal dimerization, a novel pre-N UBL domain, and the localization of human disease variants to what we hypothesize to be highly dynamic regions of the protein (i.e., interdomain and intermolecular interfaces) represent a major step forward in understanding the molecular basis of these two conditions.

## Methods

### Cloning, protein expression, and purification

Human SMCHD1 containing residues 24–580 was cloned into the multicloning site of the pGEXM vector containing a Tobacco Etch Virus (TEV) protease cleavage site between the glutathione S-transferase (GST) gene and the target using the BamHI and NotI restriction sites^37^. Point variants in *SMCHD1* were introduced by site-directed mutagenesis using overlap extension PCR and confirmed by Sanger sequencing (Genewiz). For protein expression, the vector was transformed into Rosetta2(DE3) cells (EMD Millipore). For cell growth, 20 ml of an overnight cell culture was added to 12 fernbach flasks each containing 1 L of terrific broth media with 100 µg/ml ampicillin and 35 µg/ml chloramphenicol. Cells were grown while shaking at 37 °C until an optical density of 0.8–1.0 at 600 nm was obtained at which point the temperature was set to 16 °C. Once the temperature equilibrated, 2 ml of a 0.2 M solution of IPTG was added followed by overnight expression. Cells were pelleted then resuspended in sonication buffer consisting of 25 mM Tris pH 7.5 and 500 mM NaCl. Cells were sonicated in the presence of phenylmethane sulfonyl fluoride and cOmplete EDTA-free (Roche) protease inhibitors and subsequently clarified by centrifugation. The protein was extracted from the solute by binding to Glutathione 4B Sepharose (GE) in batch. Resin was washed in sonication buffer and SMCHD1 cleaved from the resin-bound GST using TEV protease. SMCHD1 was concentrated, then loaded onto a 26/60 Superdex200 (GE) gel filtration column equilibrated in 25 mM Tris pH 7.5 and 100 mM NaCl. Wild-type and disease mutant proteins were then concentrated as needed for biochemical experiments.

For structure determination, the seleno-methionine-labeled SMCHD1 construct 24–580 with a E147A variant was expressed in B834(DE3) cells (EMD Millipore) using minimal media (100 µg/ml ampicillin and 35 µg/ml chloramphenicol) supplemented with amino acids where selenomethionine was substituted for methionine. Expression and purification were similar to above except 1 mM of dithiothreitol (DTT) was present in the sonication buffer and protein was equilibrated with 5 mM Mg^2+^/ATP prior to running on the 26/60 Superdex200 which was pre-equilibrated in 25 mM Tris pH 7.5, 100 mM NaCl and 1 mM Mg^2+^/ATP. This produced two peaks corresponding to dimer and monomer. For crystallization experiments, the dimer peak was further concentrated, and buffer exchanged into 25 mM Tris pH 7.5 and 50 mM NaCl (buffer A) and then loaded on to a MonoQ 5/50 GL (GE) column equilibrated in buffer A. Protein was eluted with a gradient of 25 mM Tris pH 7.5 250 mM NaCl. Isolated protein was dialyzed against 25 mM Tris pH 7.5, 100 mM NaCl, and 1 mM DTT then concentrated.

### Crystallization and structure determination

Crystals of seleno-methionine-labeled SMCHD1 E147A were grown from protein at 8.5 mg/ml in the presence of 5 mM Mg^2+^/ATP. Protein was crystallized using the sitting drop vapor diffusion technique with a drop consisting of 400 μl protein and 300 μl reservoir consisting of 25 mM Bis-Tris-Propane pH 6.8, 50 mM ammonium sulfate, 5% glycerol, 7.5% PEG 8000, and 5 mM DTT. For data collection, crystals were transferred to a solution consisting of 40 mM Tris pH 6.8, 80 mM ammonium sulfate, 8% glycerol, 12% PEG 8000, 19% ethylene glycol, and 0.5 mM Mg^2+^/ATP, then flash frozen in liquid nitrogen. Data were collected in four scans on a single crystal at different positions at the Southeast Regional Collaborative Access Team (SER-CAT) 22-ID beamline at the Advanced Photon Source, Argonne National Laboratory with a wavelength of 0.97625 Å and the structure was solved using single-wavelength anomalous dispersion (SAD) phasing. Data were processed using HKL2000 software using the Auto Correction function to output only reflection with informative anomalous signal (completeness of data after Auto Correction was 86.7% overall and 57.4% last shell)^38^. Using this data set, 19 selenium positions were determined with SHELX, phases calculated with mlphare, and partial models built with ARP/wARP and Buccaneer within HKL3000^39–42^. After initial model building, reflection data were reprocessed in HKL3000 without the use of Auto Correction excluding frames that did not appear to merge well while maintaining high redundancy and completeness. Further model building was carried out with iterative cycles of manual model building in Coot and refined using Phenix^43,44^. The quality of the structure was assessed using Molprobity (Table 1)^45^. The asymmetric unit consisted of 2 homodimers of SMCHD1 with each monomer containing 1 molecule of ATP. All structural figures were generated using PyMOL, with the exception of Supplementary Fig. 3 where Chimera was used^46,47^.

### Analytical chromatography

Analytical gel filtration experiments to determine oligomeric states of the protein were performed using a 10/300 Superdex200 increase column (GE). SMCHD1 constructs were loaded on to the column using 100 µl protein at 5 mg/ml in 25 mM Tris pH 7.5 and 100 mM NaCl with and without incubating in the presence of 5 mM Mg^2+^/ATP for 25 min on ice. For samples run in the absence of Mg^2+^/ATP, a running buffer of 25 mM Tris pH 7.5 and 100 mM NaCl was used. For samples run in the presence of Mg^2+^/ATP, 1 mM MgCl_2_ and 1 mM ATP was added to the running buffer. Gel filtration standards from BioRad were used to determine the molecular weight of the protein (Standards: Thyroglobulin (bovine), 670,000; γ-globulin (bovine), 158,000; Ovalbumin (chicken), 44,000; Myoglobin (horse), 17,000; and Vitamin B12, 1,350).

### Differential scanning fluorimetry

Thermal melt experiments were performed using the Melt Curve application on a QuanStudio7Flex system (Applied Biosystems). Reactions were performed in triplicate in 20 μl volumes consisting of 25 mM Tris pH 7.5, 100 mM NaCl with 0.5 mg/ml protein, and 1000-fold dilution of stock 5000X SYPRO Orange (Molecular Probes). Two biological replicates were collected in triplicate for the wild-type protein. In all, 1315 data points were collected from 25 °C to 95 °C with a ramp rate of 0.05 °C/s using an emission filter at 586 nm and an excitation filter at 470 nm. Data were analyzed using the Protein Thermal Shift Software v1.3 (Applied Biosystems) with Tm being calculated from the derivative of the melt curve.

### ATP hydrolysis assays

The radiometric ATPase assay was performed with wild-type SMCHD1 (24–580, 24–406, and 110–580) constructs and mutant SMCHD1 (24–580) constructs. Each ATPase reaction (10 μl) contained 20 mM HEPES pH 7.5, 1 mM MgCl_2_, 0.1 mg/ml BSA, 1 mM DTT, and 3.84 μM of SMCHD1; reaction was started by adding 100 nM ATP and 1 uCi of [γ-32P] ATP as a tracer and incubated at 37 °C. Reaction was stopped after 60 min by adding 2.5 μl of 500 mM EDTA pH8.0. From each stopped reaction, 1 μl was spotted onto a PEI-cellulose plate (Sigma), air-dried, and developed by thin-layer chromatography (TLC) with 1 M formic acid, and 0.5 M lithium chloride. The TLC plate was then air-dried and exposed to a storage phosphor screen. The screen was scanned by Typhoon (GE) and the intensity of the [γ-32P] ATP and the released inorganic phosphate (32 Pi) spots were quantified by ImageQuant. After subtracting background, released Pi (μΜ) was calculated from the ratio of hydrolyzed ATP (Pi) versus total 32P (ATP plus Pi) for each time point and plotted versus time (min). Experiments included at least two biological replicates and three technical replicates.

### K_m_ determination

Colorimetric ATPase activity of wild-type and mutant SMCHD1(24–580) constructs was determined using the _Pi_ColorLock Gold Kit (Innova Biosciences) which detects the amount of free _Pi_ bound to malachite green dye. Each reaction mixture (50 μl) contained 20 mM HEPES, pH 7.5, 1 mM MgCl_2_, 0.1 mg/ml BSA, 1 mM DTT, and 3.84 μM purified protein and one of five ATP concentrations (0.2, 0.5, 1, 2, or 5 mM). Reactions were incubated at 37 **°**C for 10 min, a time point determined to be in the linear range. Reactions were quenched with 100 mM EDTA then transferred to a 96-well microplate. Dye reagent was added and incubated for 5 min prior to the addition of stabilizer then incubated for another 30 min. P_i_ release was calorimetrically measured at 635 nm and phosphate standards in the kit were used to generate a standard curve. The manufacturer’s instructions were followed to calculate the amount of P_i_ in each reaction. Curve fitting was performed with the GraphPad Prism 6 Software using the Michaelis–Menten model.

### Protein dimerization

Blue-native PAGE was performed using the NativePAGE 4–16% Bis-Tris Gel (Invitrogen). Protein samples were incubated in the presence and absence of 5 mM MgCl2 and ATP in 6.5 μl of reaction mixture with 2.2 μg/μl of protein for 30 min on ice. Samples were then mixed with 2.5 μl of NativePAGE™ Sample Buffer (4×) and 1 μl of NativePAGE™ 5% G-250 Sample Additive and 10 μg total protein was applied onto each well. Gels were run with 1X NativePAGE™ anode Running Buffer and Dark Blue Cathode Buffer (contains 0.02% G-250) containing 1 mM MgCl2 and 1 mM ATP on ice at 150 V for 6–7 h. Proteins were fixed with a 40% methanol and 10% acetic acid solution and de-stained by 8% acetic acid to visualize protein bands. Gels were scanned at the 700 nm channel for 2 min to quantify monomer and dimer bands (ImageStudio, LI-COR Biosciences).

A glutaraldehyde-mediated crosslinking assay was performed as previously described^27^ but with slight modification. SMCHD1 protein (24–580, 110–580, or 24–406) was diluted to 2 mg/mL in a buffer containing 20 mM HEPES pH 7.5, 100 mM NaCl, and 10% glycerol with or without 5 mM MgCl_2_ and 5 mM ATP and incubated for 40 min at room temperature. The sample was diluted 6.25-fold in reaction buffer and glutaraldehyde was added to a final 0.0125% in 10 μL. After 20 min, the reaction was quenched by adding 2.5 μL 2 M glycine. Monomer and dimer bands were then separated by SDS-PAGE (NuPAGE, 4–12% Bis Tris Gel) and protein bands were stained with SimplyBlue SafeStain (ThermoFisher). Gels were scanned as above with ImageStudio.

### Statistics

Dimerization and ATPase assays were performed with at least two independent experiments each with technical duplicates (dimerization) or triplicates (ATPase). Data are reported as mean ± s.d unless noted otherwise. Wild-type and mutant activity were compared using one-way ANOVA. No statistical methods were used to predetermine sample size; sample size was determined based on best practices in the field. The experiments were not randomized, and the investigators were not blinded to allocation during experiments and outcome assessment.

### Reporting summary

Further information on research design is available in the Nature Research Reporting Summary linked to this article.

## Supplementary information

Supplementary Information

Description of Additional Supplementary Files

Supplementary Data

Reporting Summary

## Data Availability

The crystal structure reported in this work has been deposited in the Protein Data Bank and assigned the PDB ID code 6MW7. All other data that support the findings of this study are available in the Supplemental materials or from the corresponding author upon reasonable request.
