# Supplementary material for: A ubiquitin-like domain is required for stabilizing the N-terminal ATPase module of human SMCHD1
Source: Commun Biol. 2019 Jul 10;2:255. doi: 10.1038/s42003-019-0499-y (PMC6620310; doi:10.1038/s42003-019-0499-y)
Supplement: Supplementary file 1 — Supplementary Information [file 42003_2019_499_MOESM1_ESM.pdf]

Supplementary Table 1. Differential scanning fluorimetry results of selected SMCHD1 mutants and wild-type (wt)

Protein	Tm	ΔTm (Mut-wt)
wt	43.7 ± 0.1[a]	
S135C	44.1 ± 0.0	0.4 ± 0.1[b]
N139H	42.8 ± 0.1	-0.9 ± 0.1
Q345R	44.2 ± 0.1	0.5 ± 0.1
H348R	41.2 ± 0.1	-2.5 ± 0.1
T523K	42.5 ± 0.2	-1.2 ± 0.1
E473Q	42.5 ± 0.1	-1.2 ± 0.2
E473G	38.8 ± 0.1	-4.9 ± 0.1
G137E	42.7 ± 0.1	-1.0± 0.1
L194F	40.9 ± 0.1	-2.8 ± 0.1
Y283C	43.9 ± 0.3	0.2 ± 0.3
G478E	41.4 ± 0.1	-2.3 ± 0.1

Tm = melting temperature as determined by the peak in the derivative curve

ΔTm = Tm of mutant – Tm of wildtype(wt) construct

a) ± standard deviation (stdev) based on 6 measurements for wt and 3 for mutants.

b) error propagation of standard deviation = $(stdev(Mut)^2 + stdev(wt)^2)^{1/2}$

Supplementary Table 2. Enzyme kinetic values for wild-type (WT) and selected mutant SMCHD1(24-580) constructs

SMCHD1	V_{max} (μM P_i $min^{-1}\mu M^{-1}$)	K_{m}^ (mM)	k_{cat} (min^{-1})
WT	0.78 ± 0.14	1.00 ± 0.50	0.25 ± 0.04
S135C	0.81 ± 0.09	0.71 ± 0.26	0.26 ± 0.03
G137E	0.61 ± 0.07*	0.50 ± 0.19	0.20 ± 0.02*
N139H	0.62 ± 0.07*	0.45 ± 0.18	0.20 ± 0.02*
Q345R	0.62 ± 0.09*	0.39 ± 0.21	0.20 ± 0.03*
H348R	0.52 ± 0.16*	0.41 ± 0.46	0.17 ± 0.05*
T523K	0.59 ± 0.12*	0.56 ± 0.39	0.18 ± 0.04*
E473Q	0.56 ± 0.10*	0.64 ± 0.38	0.18 ± 0.03*
E473G	0.51 ± 0.13*	0.89 ± 0.66	0.17 ± 0.04*

Results represent the mean ± SD from 2 independent determinations.

V_{max} = maximal reaction velocity; K_M = Michaelis-Menten constant; k_{cat} = catalyst rate constant. *Vmax and Kcat are lower than in wild-type ($p<0.05$) as determined by one-way ANOVA ($F(8,45)= 5.3$, $p<0.001$) followed by Holm-Sidak method. ^No difference in ATP binding affinity (Km; $F(8,45)=1.8$, $p=0.1$) between wild-type and mutants, as determined by one-way ANOVA.

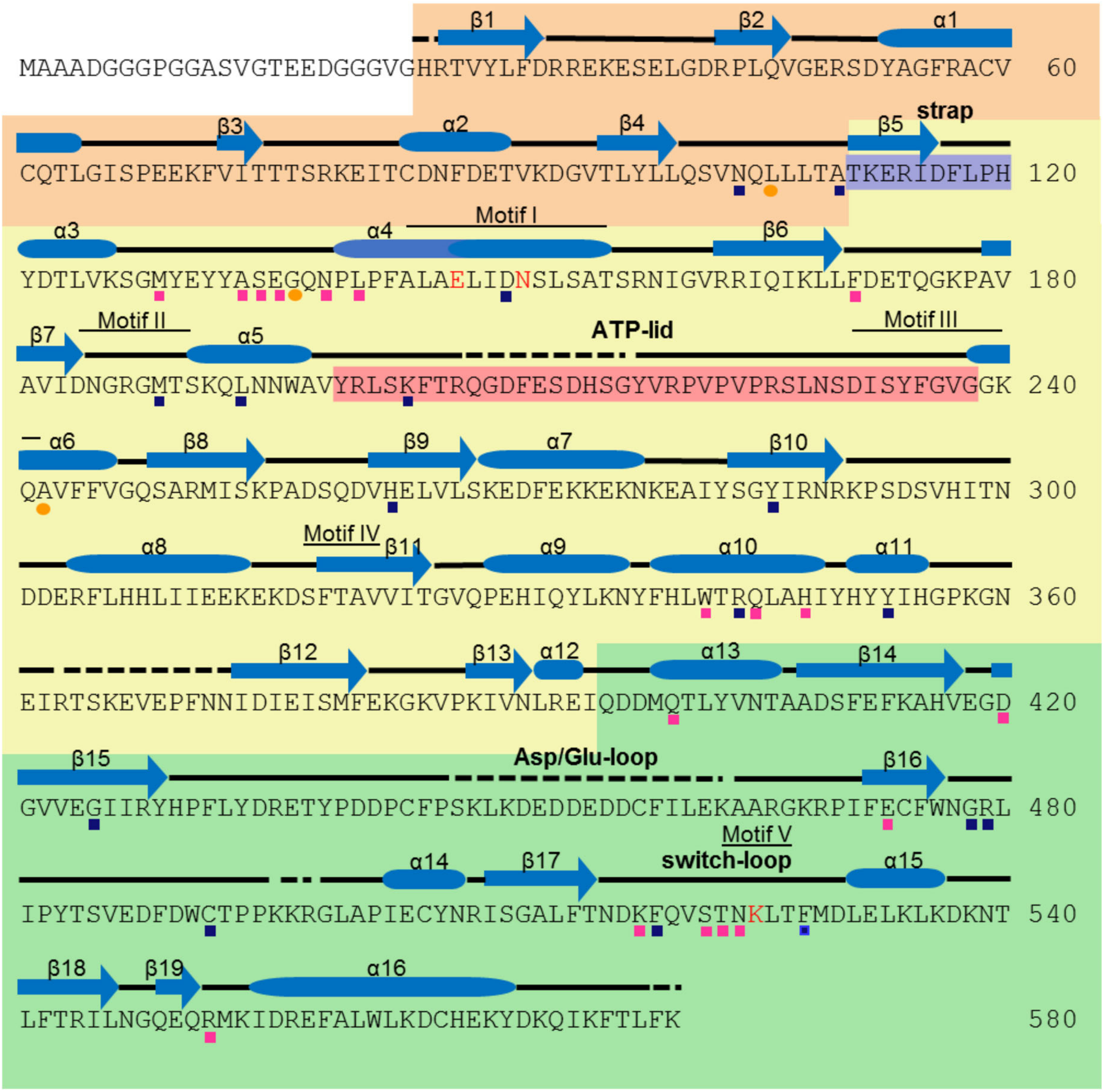

Supplementary Figure 1. Structure of the SMCHD1 ATPase module (24-580aa). **a.** Primary sequence of the SMCHD1 construct used in the current study. Residues in the pre-N ubiquitin-like (UBL) domain, GHKL-ATPase domain, and transducer domains have background colors of wheat, pale-yellow and pale-green, respectively. Secondary structural elements are labeled above the sequence with α-helices, β-sheets, and coils represented by blue ovals, blue arrows, and black lines, respectively. The black dotted line represents disordered regions in the structure. Proposed catalytic residues E147, N151 and K525 are colored red. The structural elements referred to as the “strap” and “ATP-lid” are highlighted in lavender and rose, respectively, and the five conserved GHL Motifs I-V are labeled above the corresponding sequences. Also labeled are the Asp/Glu-rich loop found near the dimer cleft and the switch-loop from the transducer domain. Pink and blue boxes below residues mark variants that have been linked to either arhinia or FSHD2, respectively, whereas orange ovals mark residues that have been implicated in both diseases.

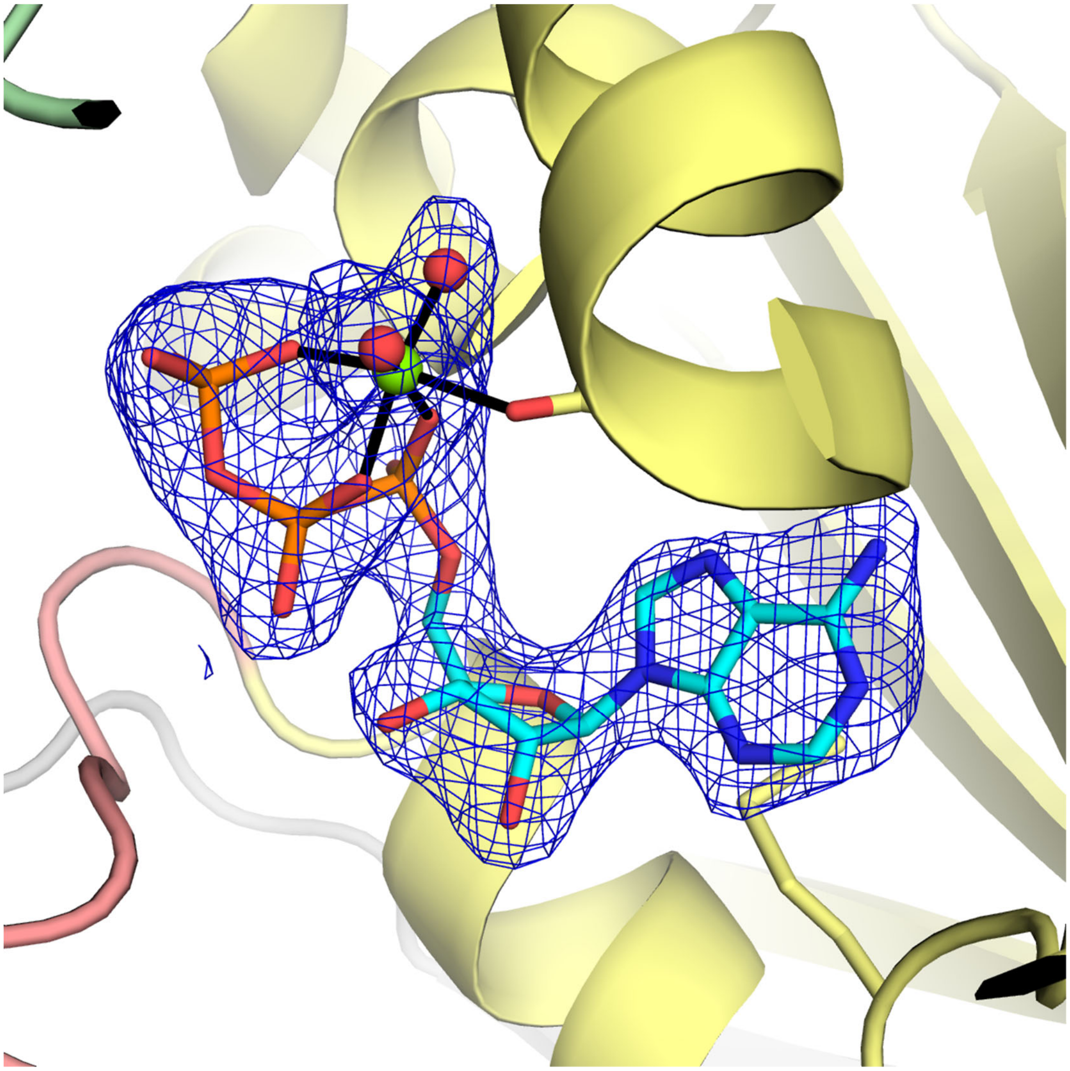

Supplementary Figure 2. Fo-Fc simulated annealing omit map of the ATP, Mg^{2+} and coordinating waters found in the active site of monomer A contoured at 3.5σ.

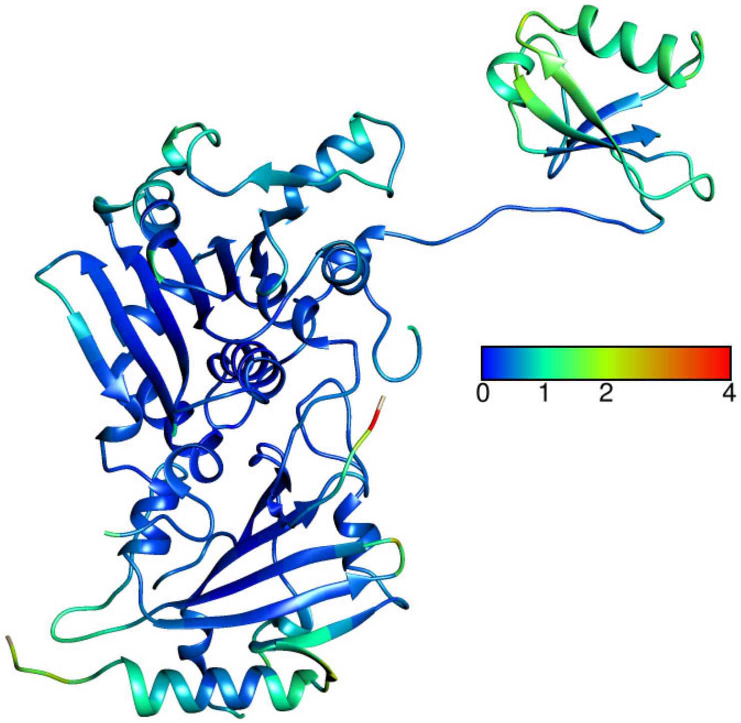

Supplementary Figure 3. Structure of monomer A from the SMCHD1 crystal structure with the RMSD between the four monomers in the asymmetric unit displayed by color from 0 Å blue to 4 Å in red.

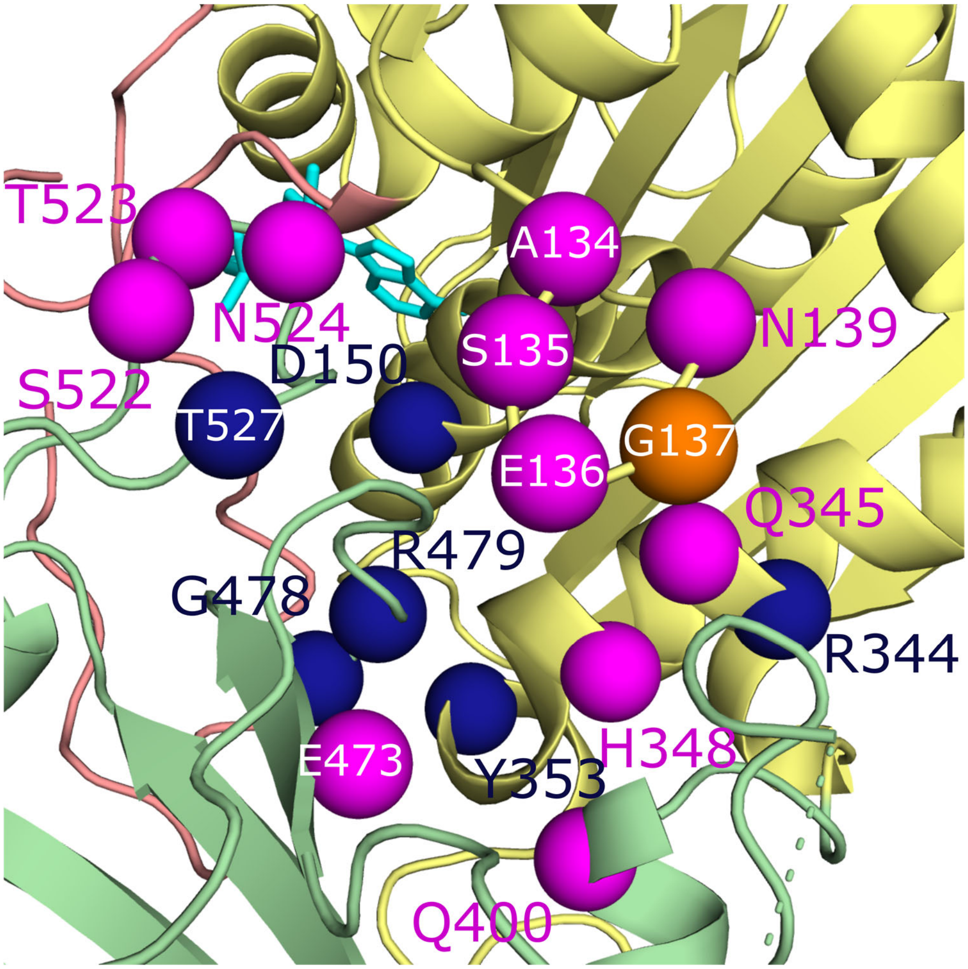

Supplementary Figure 4. Disease variant residues at dimer and interdomain interfaces. Alternate view of the position of disease related variants located near the interdomain and dimer interfaces. Variants associated with arhinia, FSHD2, or with both diseases are colored magenta, dark blue, or orange, respectively.

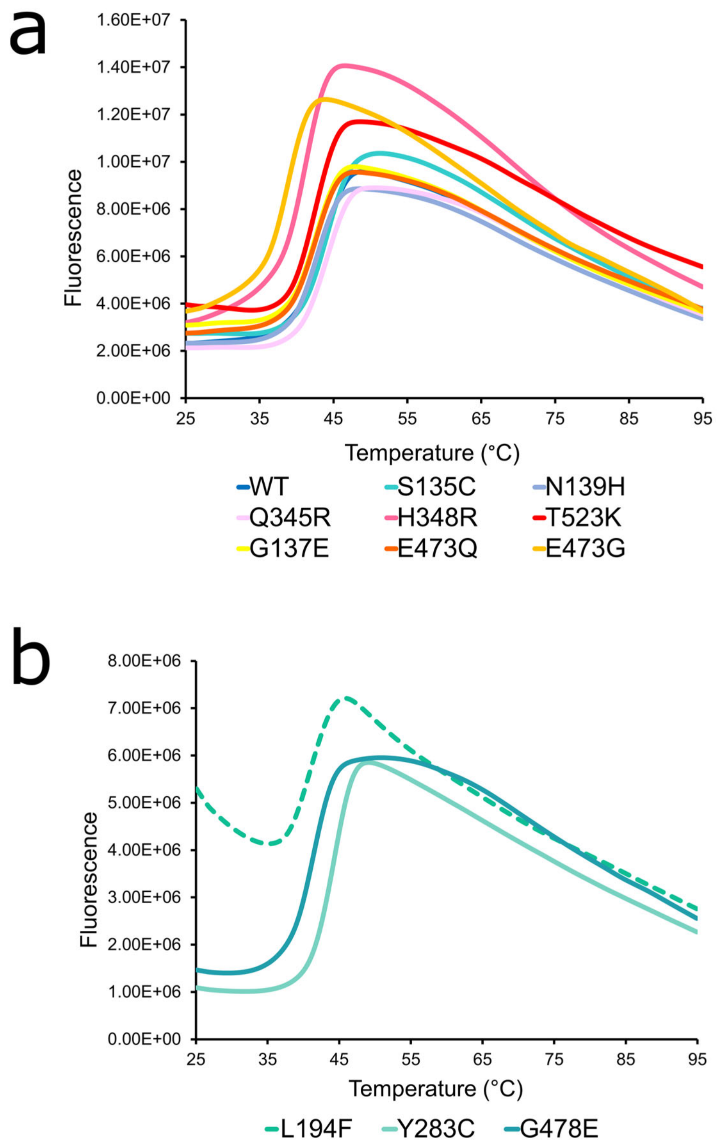

Supplementary Figure 5. Representative thermal melt curves for arhinia variants (**a**) and FSHD2-specific variants (**b**). Wild-type and G137E protein associated with both diseases are shown in (**a**). Protein was previously purified by gel filtration in the absence of ATP and eluted as monomers.

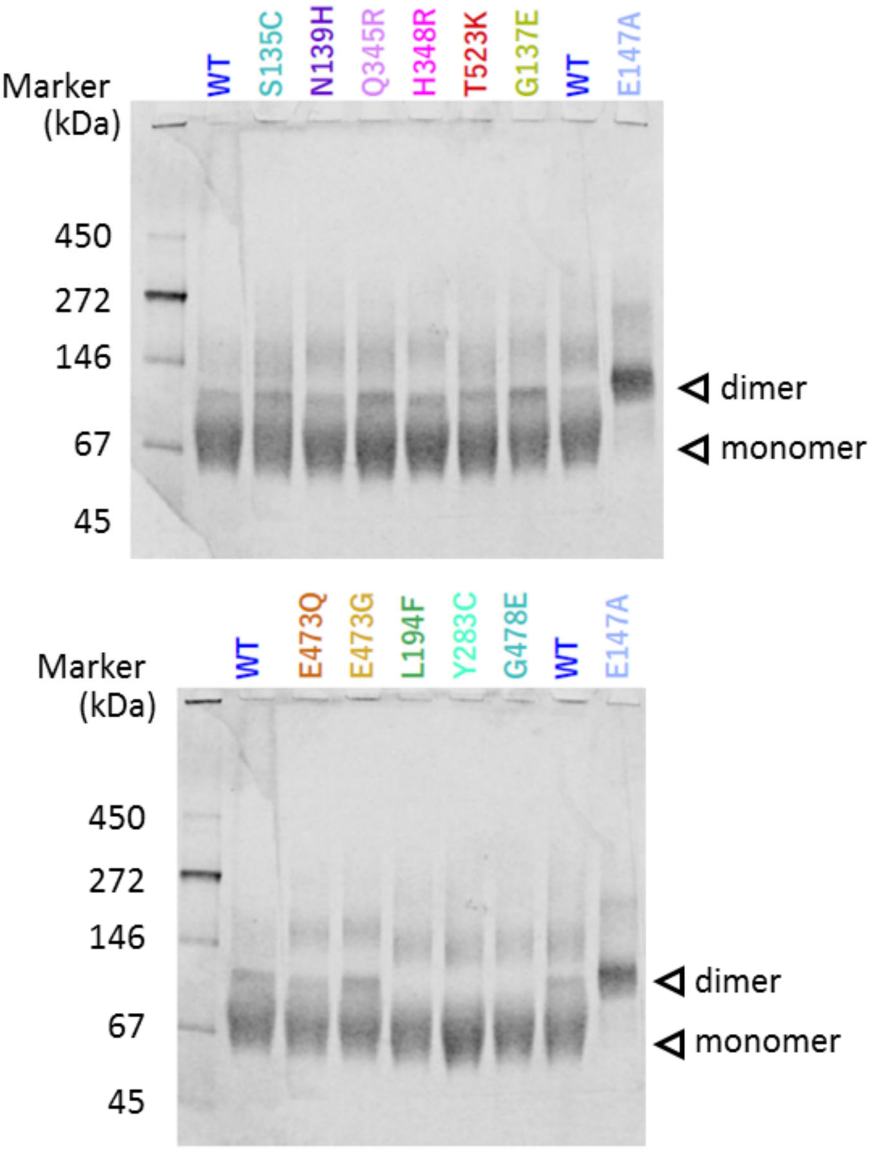

Supplementary Figure 6. Native PAGE gel demonstrating dimerization capacity of SMCHD1(24-580) wild-type and mutant constructs in the presence of ATP. Theoretical molecular weights of the monomer and dimer are 64.5 kDa and 129 kDa, respectively. Note that E147A represents the dimeric fraction after gel purification and is shown to mark the size of the dimer.

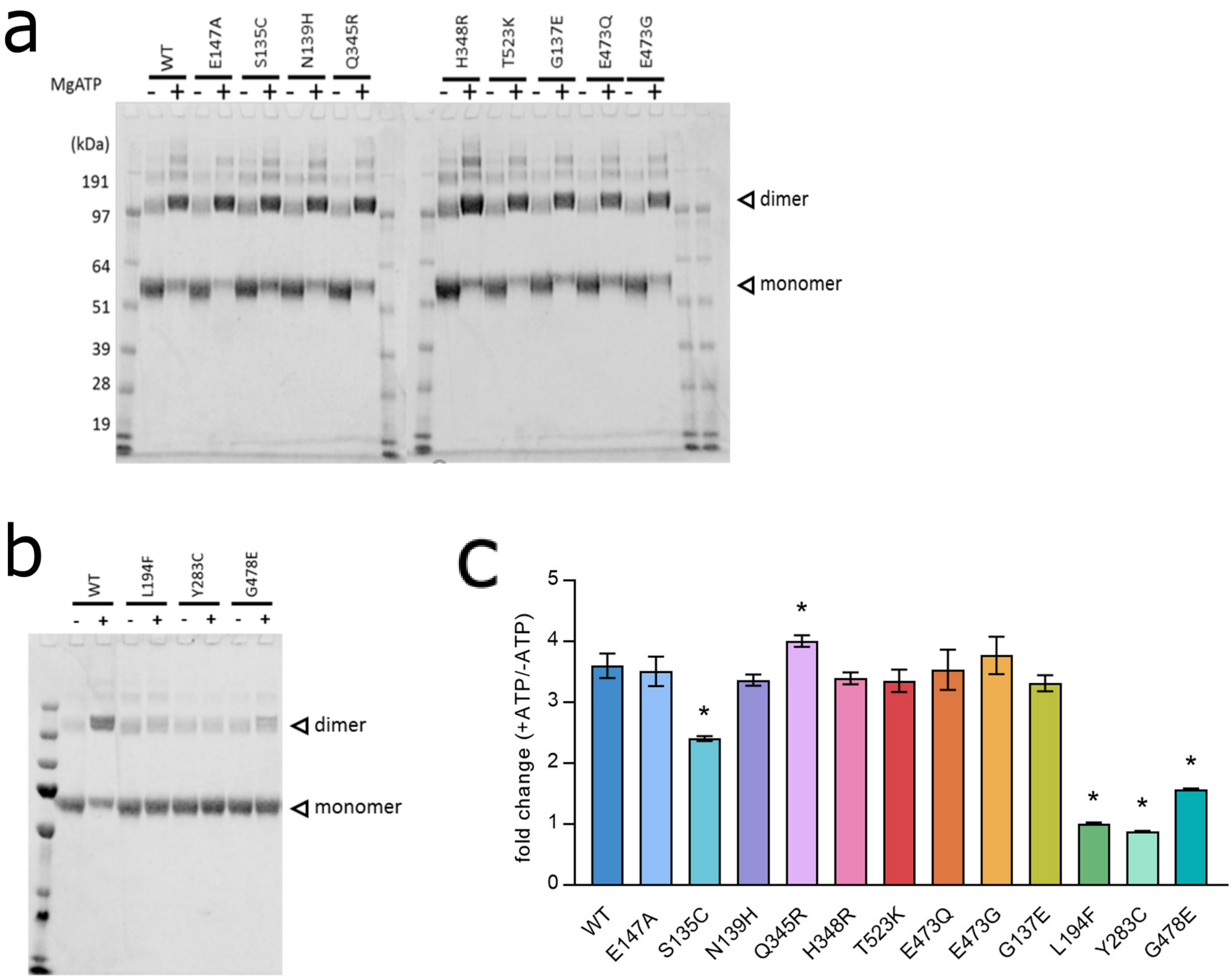

Supplementary Figure 7. Chemical cross-linking experiments in SMCHD1(24-580) with glutaraldehyde demonstrate ATP-dependent dimer formation in wild-type (WT) and arhinia mutant constructs (**a**) but not in FSHD2-specific constructs (**b**). E147A represents protein from the monomeric fraction after gel purification. Summary data (**c**) is presented as mean ± 1 SD and represents the fold-change in dimerization (dimer/[dimer + monomer]) with ATP compared to without ATP. Each experiment included 2 technical replicates and 1-2 biological replicates. *P<0.05 compared with WT.
