# Supplementary material for: A ubiquitin-like domain is required for stabilizing the N-terminal ATPase module of human SMCHD1
Source: Commun Biol. 2019 Jul 10;2:255. doi: 10.1038/s42003-019-0499-y (PMC6620310; doi:10.1038/s42003-019-0499-y)
Supplement: Supplementary file 2 — Description of Additional Supplementary Files [file 42003_2019_499_MOESM2_ESM.pdf]

Supplementary Data

Individual data points plotted in the main and supplementary figures and exact p-values for all comparisons between wild-type and mutant constructs.
